# Post-cholecystectomy Changes in the Common Bile Duct Diameter: A Comparative Ultrasound Study

**DOI:** 10.7759/cureus.100955

**Published:** 2026-01-06

**Authors:** Elma Mujakovic, Minela Becirovic, Anesa Terzic, Emir Becirovic, Dalila Kavgic, Amir Becirovic, Admir Abdic, Samir Jusupovic, Eldar Isakovic, Jasmin Delic

**Affiliations:** 1 Department of Anatomy, Faculty of Medicine, University of Tuzla, Tuzla, BIH; 2 Department of Nephrology, Internal Medicine Clinic, University Clinical Centre Tuzla, Tuzla, BIH; 3 Department of Paediatrics, Health Centre Gracanica, Gracanica, BIH; 4 Intensive Care Unit, Internal Medicine Clinic, University Clinical Centre Tuzla, Tuzla, BIH; 5 Department of Physiology, Faculty of Medicine, University of Tuzla, Tuzla, BIH; 6 Department of Endocrinology, Internal Medicine Clinic, University Clinical Centre Tuzla, Tuzla, BIH; 7 Department of Surgery, Cantonal Hospital Bihać, Bihać, BIH; 8 Department of Internal Medicine, Health Centre Kladanj, Kladanj, BIH

**Keywords:** bile duct dilatation, biliary anatomy, cholecystectomy, common bile duct, postoperative adaptation, ultrasonography

## Abstract

Background

Dilatation of the common bile duct (CBD) after cholecystectomy is frequently observed during follow-up imaging; however, its extent and clinical implications remain incompletely defined. Distinguishing physiological postoperative ductal enlargement from pathological dilatation is essential to avoid unnecessary diagnostic evaluation. This study aimed to compare CBD diameter in post-cholecystectomy patients with non-operated controls and to assess its association with time since surgery, age, and body mass index (BMI).

Materials and methods

This retrospective observational study included 165 adult patients who underwent abdominal ultrasound examination, comprising 91 post-cholecystectomy patients and 74 controls with an intact gallbladder. The CBD diameter was measured in the suprahilar segment. Group differences were evaluated using independent t-tests and chi-square tests. Logistic and linear regression analyses were used to assess predictors of CBD dilatation and continuous diameter change. All multivariable models were adjusted for age, sex, and BMI.

Results

CBD diameter was significantly greater in post-cholecystectomy patients compared with controls (6.61 mm vs. 4.56 mm; p < 0.001). Dilatation ≥7 mm occurred in 38.5% of post-cholecystectomy patients versus 5.4% of controls (p < 0.001), and prior cholecystectomy remained a strong independent predictor of dilatation after adjustment (aOR = 14.583; 95% CI: 4.449-47.807). Using a fixed ≥7 mm cutoff, increasing age was associated with lower odds of categorical CBD dilatation, whereas sex and BMI were not significant predictors. Linear regression analyses demonstrated a significant positive association between CBD diameter and both time elapsed since surgery and age, indicating gradual ductal enlargement over time. Marked dilatation (>10 mm) was uncommon and did not reach statistical significance in relation to cholecystectomy.

Conclusion

Cholecystectomy is associated with measurable and progressive enlargement of the CBD. While CBD diameter increases gradually with advancing age and postoperative duration, categorical dilation thresholds are more strongly influenced by surgical status than by age alone. Recognition of this expected postoperative anatomical pattern may help clinicians avoid unnecessary imaging and interventions in asymptomatic patients.

## Introduction

The common bile duct (CBD) is a key component of the biliary system. It serves as the main conduit for bile drainage from the liver and gallbladder into the duodenum through the ampulla of Vater. Anatomically, it lies within the hepatoduodenal ligament and runs alongside the portal vein and the proper hepatic artery, forming the portal triad [1]. The diameter of the CBD varies physiologically with sphincteric tone, bile flow, and prior surgical interventions, making its accurate assessment essential in both anatomical and clinical settings [2,3].

In clinical practice, the assessment of CBD diameter remains crucial for distinguishing physiological variations from pathological dilatation. Reference ranges for ultrasonography differ among studies and generally range from 5 to 9 mm, with upper limits of approximately 7-8 mm in individuals with an intact biliary system [4]. After cholecystectomy, the CBD may widen over time as the biliary system adapts to the absence of the gallbladder’s storage function, and diameters up to 10 mm can still be considered physiological in this setting, particularly in older adults [5,6]. Ultrasonography, due to its non-invasive nature and wide availability, remains the preferred method for evaluating these anatomical and functional changes.

Although postoperative changes of the CBD have been extensively investigated, the reported findings remain inconsistent and, at times, contradictory, preventing a clear consensus. Several studies have demonstrated postoperative widening of the CBD [6], whereas others have reported no measurable morphometric change [7,8]. A review by Kratzer et al. [9] summarised these discrepancies, emphasising that although CBD diameter most often increases after cholecystectomy, most values remain within reference limits and lack clear clinical significance. Furthermore, no uniform cutoff exists for defining clinically relevant postoperative dilatation. While some authors consider 7 mm the upper limit of normal, others suggest 8 mm, and values exceeding 10 mm are most often regarded as pathological [10,11].

Nevertheless, the degree and mechanisms of CBD enlargement after cholecystectomy remain incompletely understood. Previous studies have demonstrated inconsistent associations between CBD diameter and factors such as time since surgery, age, and BMI [12,13]. Proposed anatomical and morphometric adaptations, including altered biliary pressure dynamics, sphincter behaviour, and duct wall compliance, also remain insufficiently defined, particularly across different patient populations [8]. From a clinical perspective, differentiating expected postoperative widening from obstructive pathology is essential to avoid unnecessary diagnostic procedures. In everyday practice, ultrasonography represents the first-line imaging modality due to its accessibility and non-invasiveness. At the same time, magnetic resonance cholangiopancreatography (MRCP) and endoscopic retrograde cholangiopancreatography (ERCP) are reserved for selected patients with persistent diagnostic uncertainty or concerning clinical features [11].

Therefore, this study aimed to quantitatively analyse CBD diameter in individuals after cholecystectomy compared with non-operated controls and to evaluate its correlation with time elapsed since surgery, age, and BMI. We hypothesised that anatomical enlargement of the CBD diameter progressively increases over time following cholecystectomy, with older age and higher BMI contributing to gradual ductal widening.

## Materials and methods

Study design and population

This retrospective observational comparative study was conducted between March 1 and August 31, 2025, at the Department of Radiology at the Health Centre Gracanica. The study analysed previously recorded medical documentation and routinely performed abdominal ultrasound examinations from the study period, without any additional diagnostic or therapeutic interventions. Ethical approval was obtained from the Ethics Committee of the Health Centre Gracanica, Gracanica, Bosnia and Herzegovina (approval number: 02-335-2/25). All data were analysed in anonymised form. A total of 165 adult participants were included in the analysis, comprising 91 subjects in the post-cholecystectomy group and 74 subjects in the control group without prior biliary surgery. All consecutive cases examined during the study period that fulfilled the eligibility criteria were included.

Inclusion and exclusion criteria

The study included adult patients with clearly visualised hepatobiliary anatomy on abdominal ultrasound examination. Both post-cholecystectomy and control participants were recruited from the outpatients department. Ultrasound examinations were performed either as part of postoperative follow-up, periodic systematic health evaluations, or routine clinical assessment, with or without non-specific abdominal complaints. Subjects in the control group were selected among individuals without previous hepatobiliary or pancreatic surgery and without a history of liver or biliary disease. In the post-cholecystectomy group, documented information regarding the time elapsed since surgery was mandatory. Patients were excluded if they had chronic liver or pancreatic disorders, obstructive biliary pathology such as choledocholithiasis or biliary strictures, acute cholangitis, biliary or pancreatic malignancy, prior hepatobiliary operations other than cholecystectomy, previous biliary endoscopic or surgical interventions, significant postoperative complications, or if technical limitations prevented adequate visualisation of the common bile duct.

Ultrasound technique and measurement protocol

All examinations were performed using a GE Vivid T9 (v206) ultrasound system (GE Medical Systems China Co., Ltd., China, CE 0197 certified), equipped with a 3-5 MHz convex transducer suitable for abdominal imaging. Patients were examined in the supine position during quiet respiration, using both subcostal and intercostal approaches to ensure adequate visualisation of the hepatobiliary system. The CBD was identified within the hepatoduodenal ligament, adjacent to the portal vein and the proper hepatic artery. Its luminal diameter was measured using the inner-to-inner method in the suprahilar segment, just distal to the confluence of the cystic duct and common hepatic duct. Each measurement was obtained from a frozen image at maximal clarity. The same experienced sonographer performed all ultrasound examinations to minimise interobserver variability.

Data collection and study variables

For each participant, demographic and clinical data were collected, including age, sex, body weight, height, BMI, cholecystectomy status, and, for the post-cholecystectomy group, the time elapsed since surgery. The primary outcome variable was the diameter of the CBD, measured in millimetres. Secondary variables included sex, BMI, and duration since cholecystectomy. For categorical analyses, CBD diameters were classified into four ranges: ≤5 mm, 5.1-6.9 mm, 7-10 mm, and >10 mm, in accordance with values commonly reported in the literature.

Statistical analysis

Statistical analysis was performed using IBM SPSS Statistics for Windows, version 26.0 (IBM Corp., Armonk, NY, USA). Continuous variables were assessed for normality using visual inspection and distribution testing and are presented as mean values with 95% confidence intervals, while categorical variables are expressed as frequencies and percentages. Comparisons between the post-cholecystectomy and control groups were performed using the independent-samples t-test for continuous variables and the chi-square test for categorical variables. The distribution of CBD diameter categories between groups was also analysed using the chi-square test. Multivariable logistic regression analyses were performed to identify independent predictors of CBD dilatation using two dichotomous outcomes: CBD≥7 mm and CBD>10 mm. The models included cholecystectomy status, age, sex, and BMI as covariates and are reported as adjusted odds ratios with 95% confidence intervals.

Simple linear regression analysis was performed in the post-cholecystectomy group to assess the relationship between time since surgery and CBD diameter. In addition, simple linear regression analysis was conducted in the overall sample to evaluate the association between age and CBD diameter. Regression results are presented as unstandardized regression coefficients, standard errors, standardised coefficients, coefficients of determination, and F statistics. All statistical tests were two-tailed, and a p < 0.05 was considered statistically significant.

## Results

A total of 165 subjects were analysed, including 91 patients (55.2%) in the post-cholecystectomy group and 74 patients (44.8%) in the control group without prior biliary surgery. Women predominated in the overall cohort, with a significantly higher proportion of female patients in the post-cholecystectomy group compared with the control group (91.2% vs. 63.5%; p < 0.001). Apart from sex distribution, the two groups were comparable in terms of age and BMI (both p > 0.05). In contrast, the mean CBD diameter was significantly larger in the post-cholecystectomy group than in controls (6.61 vs. 4.56 mm; p < 0.001). Baseline demographic and clinical characteristics are summarised in Table 1.

**Table 1:** Baseline demographic and clinical characteristics of the study population Categorical variables were compared using the Pearson chi-square test. Continuous variables were analysed using the independent-samples t-test or the Mann-Whitney U test, as appropriate, based on data distribution. P-values were calculated using the corresponding statistical tests. BMI, body mass index; CBD, common bile duct; CI, confidence interval.

Variable	Post-cholecystectomy (n = 91)	Control group (n = 74)	Test statistic	p-value
Female sex, n (%)	83 (91.2%)	47 (63.5%)	χ² = 20.68	<0.001
Age (years), mean (95% CI)	57.20 (54.30-60.10)	59.01 (55.59-62.24)	U = 3097.5	0.337
BMI (kg/m²), mean (95% CI)	30.19 (29.25-31.14)	28.84 (27.95-29.73)	t = 0.54	0.590
CBD diameter (mm), mean (95% CI)	6.61 (6.07-7.14)	4.56 (4.20-4.97)	U = 1432.0	<0.001

**Figure 1:**
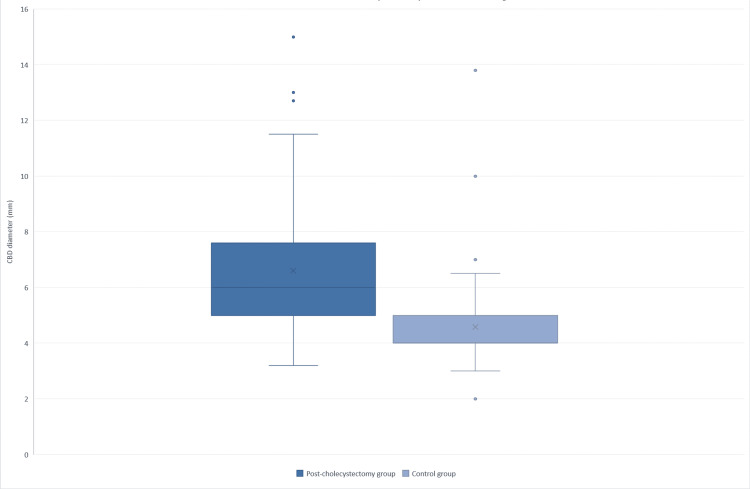
Distribution of common bile duct (CBD) diameter in post-cholecystectomy patients and the control group Box-and-whisker plot comparing common bile duct (CBD) diameter between post-cholecystectomy patients and controls. The box represents the interquartile range, the horizontal line indicates the median, and whiskers represent the minimum and maximum values excluding outliers. Post-cholecystectomy patients exhibit a higher median CBD diameter and a wider distribution than the control group.

In addition to the difference in mean CBD diameter between groups, the distribution of measurements is illustrated in Figure 1. The post-cholecystectomy group exhibited a higher median CBD diameter, a wider interquartile range, and a broader overall spread of values, including measurements exceeding 10 mm. In contrast, the control group showed a narrower distribution, with values clustered around lower diameters.

When categorising CBD diameters into clinically relevant thresholds, most control subjects had diameters ≤5 mm (77.0%), whereas only 37.4% of post-cholecystectomy patients fell within this normal range. A CBD diameter ≥7 mm was observed in 38.5% of the post-cholecystectomy group, compared with only 5.5% of controls. Severe dilatation (CBD >10 mm) remained uncommon but occurred more frequently after cholecystectomy (8.8% vs. 1.4%). The complete distribution of CBD diameter categories is presented in Table 2.

**Table 2:** Distribution of CBD diameter categories Differences in the distribution of common bile duct diameter categories between groups were assessed using the Pearson chi-square test. CBD, common bile duct.

Group	CBD ≤5 mm N (%)	CBD 5.1-6.9 mm N (%)	CBD 7-10 mm N (%)	CBD >10 mm N (%)	Total	Test statistic	p-value
Control (n=74)	57 (77.0%)	13 (17.6%)	3 (4.1%)	1 (1.4%)	74	χ²=31.35	<0.001
Post-cholecystectomy (n=91)	34 (37.4%)	22 (24.2%)	27 (29.7%)	8 (8.8%)	91		
Total (n=165)	91 (55.2%)	35 (21.2%)	30 (18.2%)	9 (5.5%)	165		

In multivariable logistic regression adjusted for age, sex, and BMI, prior cholecystectomy was the strongest independent predictor of CBD dilatation ≥7 mm (adjusted odds ratio (aOR) = 14.583, 95% CI: 4.449-47.807; p < 0.001). Increasing age was significantly associated with lower odds of categorical CBD dilatation (≥7 mm) (aOR = 0.947 per year; p = 0.003), while sex and BMI were not predictive. For dilatation exceeding 10 mm, none of the examined predictors reached statistical significance. Cholecystectomy showed a non-significant trend toward higher odds of CBD >10 mm (aOR = 7.294, 95% CI: 0.832-63.952; p = 0.073), and age demonstrated a borderline association (p = 0.081). BMI and sex did not influence extreme duct enlargement. Logistic regression results are summarised in Table 3.

**Table 3:** Logistic regression of factors associated with common bile duct (CBD) dilation (≥7 mm and >10 mm, aOR (95% CI)) P-values were calculated using Wald chi-square test within a multivariable logistic regression model adjusted for age, sex, and body mass index. aOR, adjusted odds ratio; CI, confidence interval; BMI, body mass index; CBD, common bile duct.

Variable	aOR (95% CI) for CBD 7-10 mm	Wald χ²	p-value	aOR (95% CI) for CBD > 10 mm	Wald χ²	p-value
Age (per year)	0.947 (0.913-0.981)	8.985	0.003	0.932 (0.816-1.009)	3.052	0.081
BMI (per unit)	1.023 (0.927-1.130)	0.205	0.651	0.885 (0.740-1.059)	1.774	0.183
Cholecystectomy (Yes vs No)	14.583 (4.449-47.807)	19.571	<0.001	7.294 (0.832-63.952)	3.217	0.073
Sex (Female vs Male)	0.928 (0.248-3.477)	0.012	0.912	1.112 (0.110-11.286)	0.008	0.928

Simple linear regression analysis demonstrated that, within the post-cholecystectomy group, time since surgery was a significant positive predictor of CBD diameter, with each additional postoperative year associated with a 0.122 mm increase in CBD width (B = 0.122, SE = 0.035, β = 0.350; p = 0.001). This model explained 12.3% of the variance in CBD diameter (R² = 0.123; adjusted R² = 0.113; F(1,89) = 12.442, p = 0.001). In the overall sample, age also demonstrated a significant linear association with CBD diameter (B = 0.042, SE = 0.013, β = 0.240; p = 0.002), explaining 5.8% of the CBD variance (R² = 0.058; adjusted R² = 0.052; F(1,163) = 9.960, p = 0.002). These findings, detailed in Table 4, indicate that both ageing and postoperative interval contribute to incremental enlargement of the CBD.

**Table 4:** Linear regression models predicting CBD diameter Simple linear regression analysis was used to evaluate predictors of common bile duct diameter. Statistical significance of individual regression coefficients was assessed using the t-test, while overall model significance was evaluated using the F-test. B, unstandardized regression coefficient; SE, standard error; β, standardised regression coefficient; CBD, common bile duct.

Predictor	B	SE	β	t	p-value	R²	Adjusted R²	F(df)
Time since surgery (years)	0.122	0.035	0.350	3.527	0.001	0.123	0.113	12.442 (1,89)
Age (years)	0.042	0.013	0.240	3.156	0.002	0.058	0.052	9.960 (1,163)

**Figure 2:**
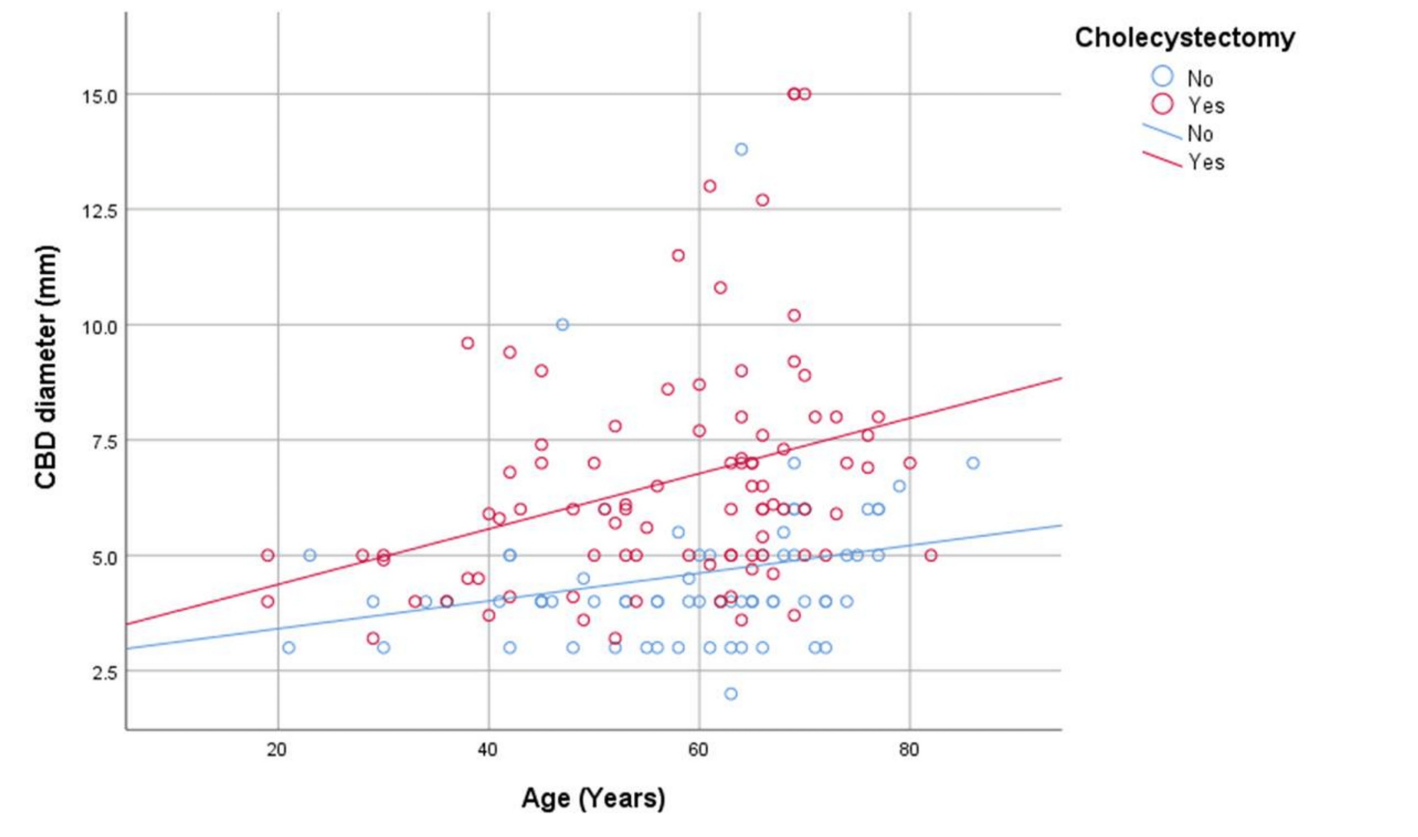
Relationship between age and common bile duct (CBD) diameter in post-cholecystectomy patients and the control group Scatter plot showing the association between age and common bile duct (CBD) diameter in post-cholecystectomy patients and the control group, with fitted linear regression lines for each group. Post-cholecystectomy patients demonstrate a steeper age-related increase in CBD diameter compared with controls, indicating a stronger association between ageing and ductal enlargement after surgery.

**Figure 3:**
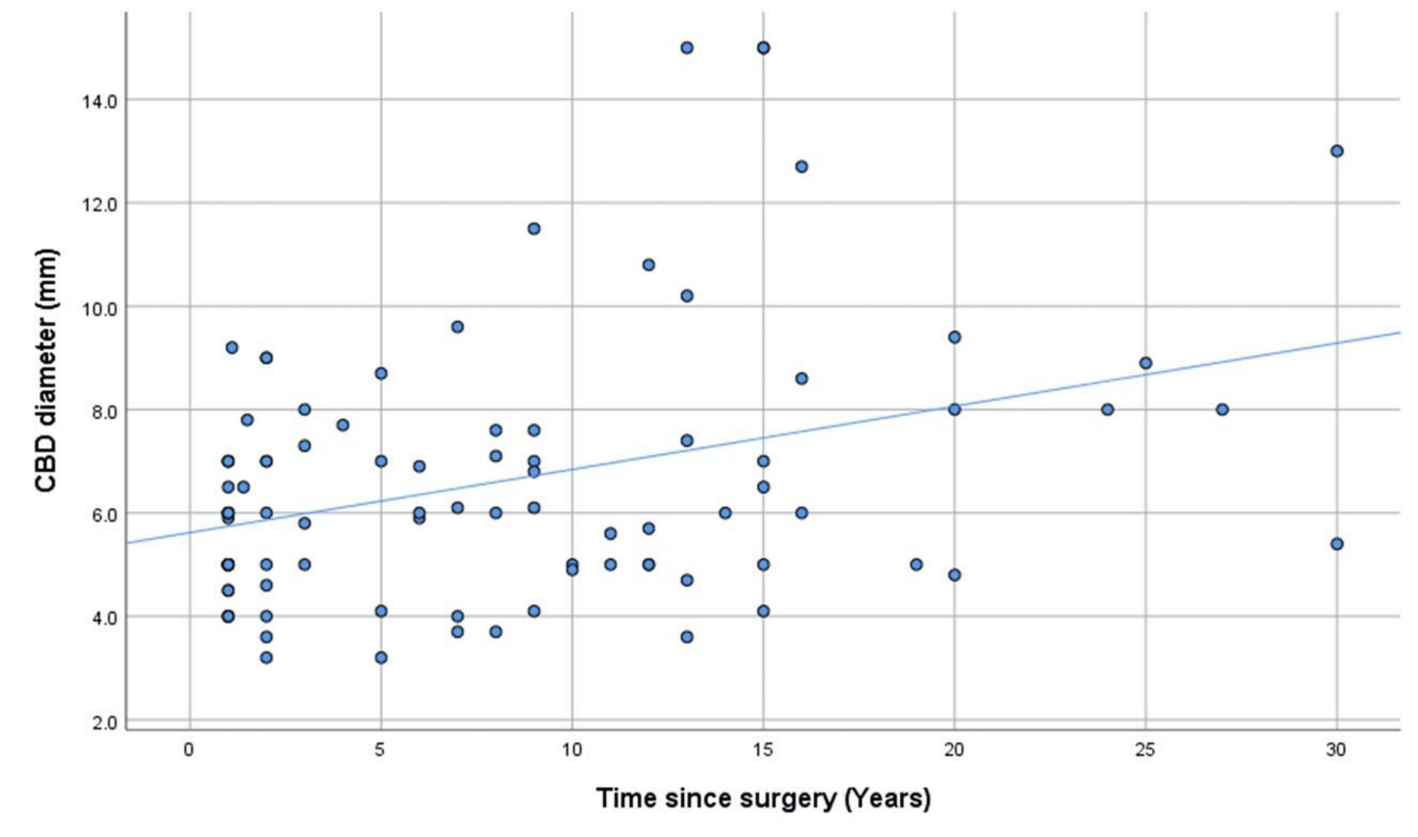
Scatter plot showing the relationship between common bile duct (CBD) diameter and time since cholecystectomy Scatter plot showing the association between time elapsed since cholecystectomy and common bile duct (CBD) diameter, with a fitted linear regression line. A significant positive relationship is observed, indicating progressive enlargement of the CBD with increasing postoperative duration.

To further illustrate these associations, scatter plots with fitted regression lines were generated. Figure 2 depicts age-related changes in CBD diameter in both groups, while Figure 3 shows the association between postoperative duration and CBD diameter in the post-cholecystectomy cohort.

## Discussion

The present study demonstrates that cholecystectomy is associated with a measurable, anatomically significant enlargement of the CBD that progresses gradually over time and with advancing age. In our cohort, patients in the post-cholecystectomy group exhibited significantly wider CBD diameters compared with controls (6.61 vs. 4.56 mm; p < 0.001), consistent with the concept of compensatory ductal remodelling following gallbladder removal [14].

From an anatomical perspective, the bile duct wall contains smooth muscle and connective tissue, allowing gradual structural adaptation [15]. The loss of the gallbladder’s reservoir function alters biliary dynamics by conversion of the biliary system into a single-outlet pathway for bile flow into the duodenum [16]. This anatomical and functional reconfiguration may also influence sphincter of Oddi activity, with reported increases in basal pressure, disordered or retrograde contractions, and loss of the normal inhibitory response to cholecystokinin [3]. As a result, mild postoperative CBD dilatation is often a physiological adaptive change, whereas marked or progressive dilatation, or dilatation accompanied by symptoms or abnormal liver tests, is more suggestive of underlying pathology [17,18].

The observed mean difference of approximately 2.0 mm in CBD diameter between post-cholecystectomy patients and controls in our study aligns well with ranges reported in previous investigations [14]. Analysis of CBD diameters according to clinically relevant thresholds revealed marked differences between the study groups. In the control group, most patients remained within the expected physiological range, with 77.0% showing CBD diameters ≤5 mm and 94.6% remaining <7 mm. After cholecystectomy, this proportion decreased substantially, with values ≥7 mm becoming more common. Although dilatation exceeding 10 mm was uncommon overall, it occurred more frequently in post-cholecystectomy patients (8.8% vs. 1.4%). These findings support prior reports indicating that while 7 mm is generally considered the upper limit of normal, diameters up to 10 mm may still represent physiological adaptation in elderly or post-cholecystectomy individuals [6,9].

Previous studies have reported wider bile duct diameters in patients with prior cholecystectomy. At the same time, larger population-based MRCP studies have further refined reference values, indicating that CBD diameters of up to 8 mm in individuals younger than 65 years and up to 11 mm in those aged 65 years or older can be considered physiological [19,20]. Our findings are entirely consistent with these observations. Nonetheless, some studies have not observed postoperative CBD dilatation, suggesting that the biliary response after cholecystectomy is variable and that ductal enlargement does not occur uniformly in all patients [21,22].

In everyday clinical practice, the important question is not simply that the bile duct may be wider after cholecystectomy, but how this should be interpreted in patients who have no clinical or biochemical signs of cholestasis. Such findings are often detected incidentally on imaging performed for unrelated reasons and, in many cases, represent a benign postoperative change [23]. Recognising this helps avoid unnecessary concern and limits additional invasive or costly investigations.

In our multivariable analysis, prior cholecystectomy emerged as the strongest independent determinant of CBD dilatation ≥7 mm, indicating a markedly increased likelihood of ductal widening. When a fixed cutoff was applied, advancing age was associated with lower odds of crossing the ≥7 mm threshold, despite a gradual increase in absolute CBD diameter with age. This apparent discrepancy likely reflects a threshold and distribution effect, in which age-related ductal widening often falls below commonly used dilation cutoffs. In contrast, sex and body mass index did not significantly influence CBD diameter, consistent with previous reports [9,24].

Linear regression analysis further supported a time-dependent widening of the CBD following surgery, with an average increase of approximately 0.12 mm per postoperative year. Although the proportion of explained variance was moderate (R² = 12.3%), this finding suggests that progressive ductal enlargement represents a gradual physiological remodelling process. A similar linear association was observed between age and CBD diameter in the overall cohort, with older age predicting wider ducts at approximately 0.04 mm per year.

These observations highlight the importance of anatomical literacy in everyday clinical practice. An accurate understanding of normal postoperative anatomy is essential, as isolated CBD widening should be interpreted in the context of age and time since surgery rather than immediately attributed to biliary obstruction [23]. Misclassification of postoperative CBD dilatation can also have important consequences. If physiological enlargement is mistaken for pathological, patients may undergo unnecessary MRCP or ERCP, with accompanying risks and healthcare costs [6]. Conversely, assuming that marked dilatation is physiological may delay the diagnosis of clinically relevant obstruction. Different diameter thresholds reported in the literature add to this uncertainty, since the same CBD value can be interpreted in different ways [4]. Therefore, postoperative CBD dilatation should always be evaluated in conjunction with symptoms, laboratory results, and the overall clinical context.

Limitations

This study has several limitations. First, it was conducted at a single centre with a moderate sample size, which may limit generalizability. Second, despite a standardised ultrasound protocol, measurement of the CBD may be influenced by operator dependence and technical factors such as bowel gas or patient positioning. Third, biochemical parameters and longer postoperative follow-up were not systematically analysed, which could have provided additional insight into the functional aspects of biliary adaptation. Finally, the cross-sectional nature of the study precludes longitudinal assessment of intra-individual ductal changes.

## Conclusions

Cholecystectomy is associated with a gradual, age-related enlargement of the common bile duct, reflecting a physiological postoperative adaptation rather than pathological change. Our findings demonstrate that this widening progresses over time and with advancing age, while clinically relevant dilation thresholds are more strongly influenced by surgical status than by age alone. Importantly, most postoperative ductal enlargement remains within non-pathological limits and should be interpreted in conjunction with clinical presentation and accompanying imaging findings. These results highlight the importance of considering both age and cholecystectomy history when evaluating CBD diameter and support the use of surgery- and age-adjusted reference values in everyday clinical practice. Future research should include prospective longitudinal studies, validation across different imaging modalities, integration of anatomical measurements with biochemical markers, and evaluation in more diverse populations to better define generalisability.
